# Complexes of DNA with fluorescent dyes are effective reagents for detection of autoimmune antibodies

**DOI:** 10.1038/s41598-017-02214-0

**Published:** 2017-05-15

**Authors:** Ivana Domljanovic, Annika Carstens, Anders Okholm, Jørgen Kjems, Christoffer Tandrup Nielsen, Niels H. H. Heegaard, Kira Astakhova

**Affiliations:** 1 Department of Physics, Chemistry and Pharmacy, University of Southern Denmark, Odense M, 5230 Denmark; 2 Interdisciplinary Nanoscience Center (iNANO), Aarhus University, Gustav Wieds Vej 14, Aarhus C, 8000 Denmark; 3 Copenhagen Lupus and Vasculitis Clinic, Centre for Rheumatology and Spine Diseases, Rigshospitalet, Copenhagen University Hospital, Copenhagen, 2100 Denmark; 4 Department of Autoimmunology and Biomarkers, Statens Serum Institute, DK-2300 Copenhagen S, Denmark; 5 Department of Clinical Biochemistry and Pharmacology, Odense University Hospital, University of Southern Denmark, DK-5000 Odense C, Denmark

## Abstract

To date, there are multiple assays developed that detect and quantify antibodies in biofluids. Nevertheless, there is still a lack of simple approaches that specifically detect autoimmune antibodies to double-stranded DNA. Herein we investigate the potential of novel nucleic acid complexes as targets for these antibodies. This is done in a simple, rapid and specific immunofluorescence assay. Specifically, employing 3D nanostructures (DNA origami), we present a new approach in the detection and study of human antibodies to DNA. We demonstrate the detection of anti-DNA antibodies that are characteristic of systemic lupus erythematosus, a chronic autoimmune disease with multiple manifestations. We tested the most potent non-covalent pairs of DNA and fluorescent dyes. Several complexes showed specific recognition of autoimmune antibodies in human samples of lupus patients using a simple one-step immunofluorescence method. This makes the novel assay developed herein a promising tool for research and point-of-care monitoring of anti-DNA antibodies. Using this method, we for the first time experimentally confirm that the disease-specific autoimmune antibodies are sensitive to the 3D structure of nucleic acids and not only to the nucleotide sequence, as was previously thought.

## Introduction

Human antibodies to nucleic acids have become ubiquitous as a tool in diagnostics and the study of autoimmune diseases^1^. This is the case in, for example, systemic lupus erythematosus (SLE)^2^. SLE is a systemic autoimmune disorder, potentially causing damage to any organ in the body via the abnormal response of the immune system to one’s own cells, tissues and biomolecules. The cause of SLE is not fully understood, but according to recent studies, anti-DNA antibodies play a crucial role by triggering the degradation of intracellular DNA after entrance into the cells^3^. Thus, in addition to anti-DNA autoantibodies being crucial for the diagnosis of SLE they are promising targets for therapy^4^. However, in spite of growing knowledge on anti-DNAs, there is still a lack of methods for their specific detection^5^.

Anti-DNAs are typically detected and quantified by immunoassays, such as enzyme-linked immunosorbent assay (ELISA) or *Crithidia luciliae* indirect immunofluorescence (IIF). Immunoassays are sensitive, versatile and simple methods that can detect and quantify targets in picomolar concentrations directly in complex biological media like serum^6, 7^. Many immunoassays can be run on very basic laboratory equipment, such as a microplate reader for ELISA^8^.

Although the assays are performed under equilibrium conditions, unfortunately they are unable to provide either any information on the structure of antigen–antibody complexes or quantitative binding characteristics^8^. Moreover, currently applied heterogeneous and unstable natural DNA molecules used as antigenic targets in these assays often result in poor reproducibility and low specificity of blood tests; around 5% of healthy persons give a weakly positive result, even though they are not suffering from SLE^2^. Detected anti-DNA antibodies also cross-react with other antigens such as phospholipid cardiolipin^2^.

DNA binds to antibodies through hydrogen bonds, van der Waals and electrostatic forces^8^. Hydrophobic contacts, together with the ion dipole bonds, contribute to the stability of protein-nucleic acid complexes, whereas hydrogen bonds with base edges are important for specificity^9^. Recently, we and others applied a computational approach to improve the understanding of DNA-antibody interactions^9, 10^. Y. An *et al*.^10^ showed that the monoclonal antibody ED-10^11^ interacts with two adjacent nucleotides in its binding site and favours dTdC over other nucleotides and that this recognition motif is highly prevalent in the polyclonal antibody species such as those present in SLE sera.

Besides antibodies, DNA uses similar types of interactions for binding to small molecules such as fluorescent dyes^12^. To date there is a plethora of fluorophores developed that bind DNA in a sequence-independent fashion. They share a similar structural motif of aromatic core that intercalates into the dsDNA and additional ‘arms’ that form stabilizing hydrogen bonds with the grooves^13^. Examples of this type include ethidium bromide, thiazole orange and acridine yellow. Another type of structure is presented by groove-binding dyes, such as Sybr Green and the recently developed analogue Eva Green^14^. Upon binding to DNA, the fluorescence of these dyes lights up 20-fold for Sybr Green and up to 130-fold for Eva Green. The light-up occurs due to the elimination of the quenching interactions of aromatic fluorophores with aqueous media when the dye is positioned within the stack and/or hydrophobic dsDNA grooves^15^. Besides high brightness, Eva Green has the advantage of low toxicity and therefore is an attractive dye for research and clinical diagnostics of dsDNA^14^.

**Figure 1:**
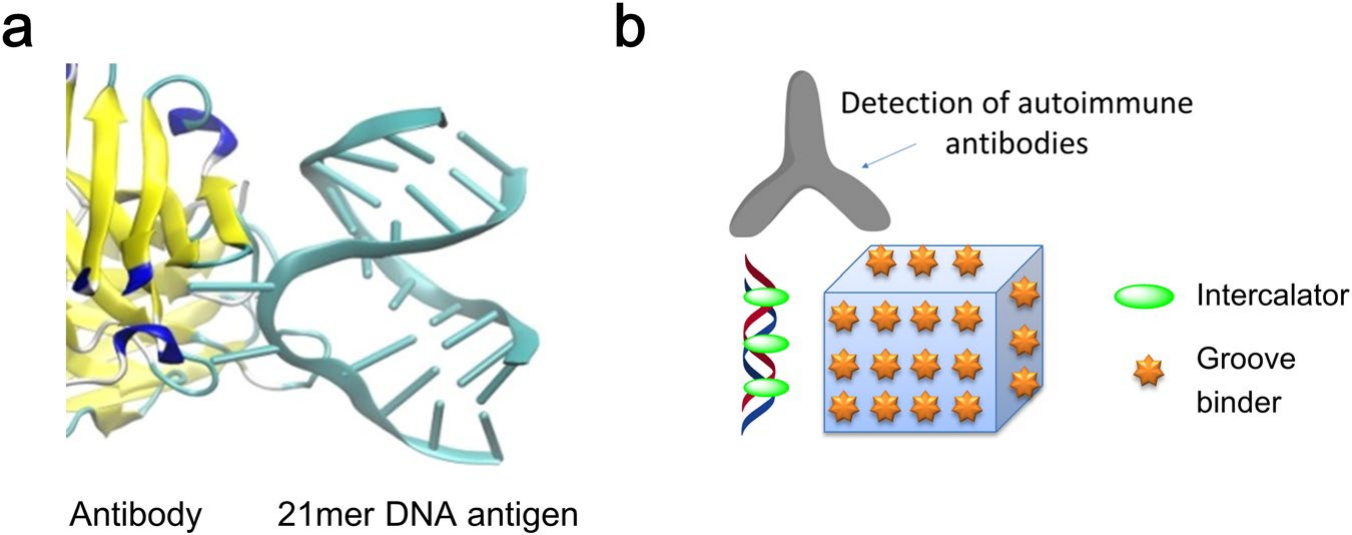
(**a**) Molecular model for the interaction between dsDNA and monoclonal autoimmune antibody ED-10^9–11^; (**b**) Two types of the synthetic antigens used in this work: short synthetic DNA and 3D DNA origami.

Valuable structural information on a-DNAs can be gained through the use of sequence-defined synthetic antigens. Synthetic oligonucleotides may be produced with high purity, good specificity and affinity, and provide well-controlled chemical structures, which make them a promising tool for diagnostics and studies of autoimmune diseases where aberrant anti-DNA immunoreactivity occurs^9^. Recently we and others proved that rational design and the incorporation of modified nucleotides into oligonucleotides can provide valuable dsDNA antigens for ELISA of a-DNA^9^. Taking these recent works into account, DNA antigens can be divided into two classes: short synthetic DNA and large, mixmer biological structures. In most studies including our recent publication, both classes are used in indirect assay (Fig. 1). However the potential of using non-covalent complexes between these synthetic antigens and the aforementioned fluorophores as reagents for the detection and study of autoimmune antibodies has not been explored thus far.

In this work we aimed at studying synthetic DNA antigens of two types, short dsDNA and large DNA origami, in a direct immunofluorescence assay (Fig. 1). Our hypothesis was that the specific recognition of DNA by corresponding antibodies displaces the number of fluorophores bound to it, which can be followed simply by fluorometry. We show that antibodies to DNA are sensitive to nucleotide composition and chemical modification in short synthetic antigens. Our study also proves that DNA origami and Eva Green dye form ultra-bright non-covalent complexes that are useful in sensing a-DNAs in lupus sera. Having studied different origami structures, our data suggest that the 3D structure of DNA origami has an effect on the recognition of antibodies. In this study also we demonstrate that the novel immunofluorescence assay detects antibodies specific to SLE, enabling simple, time- and cost-effective diagnostics of clinically relevant antibodies.

## Results

### Patient samples used in this work: selection and characterization

For this work, we selected sera samples from 28 adults who were diagnosed with SLE (SSI, Denmark). SLE samples 1–8 have been used in our recent study^9^, whereas samples SLE 9–28 were from a new cohort. To investigate specificity of antibody detection we additionally chose monoclonal and polyclonal antibodies, and patient samples with a different autoimmune disease, rheumatoid arthritis (RA, samples 1–20, provided by SSI, Denmark). RA and SLE patients fulfilled the relevant American College of Rheumatology disease criteria^16, 17^. 57% SLE (SSI) were ANA positive.

For specificity studies, we included additional ANA positive SLE samples^18^ (n = 30; 100% ANA positive) which were analysed along with non-matched healthy control cohort from SSI (n = 20) and Odense University Hospital (OUH; n = 60), and non-matched disease-stated sera from OUH: Crohn’s disease^19^ (n = 10), RA (n = 30), antiphospholipid syndrome^20^ (APS, n = 30), hepatitis B^21^ (n = 4), and cancer^22, 23^ (n = 8). Rationale for choosing these diseases as controls is that Crohn’s disease, RA and APS are autoimmune, whereas hepatitis B and cancer might raise a-DNA levels as well^16–21, 24, 25^. For the diagnosis establishment, patients fulfilled relevant disease criteria^19–23^.

All the samples have been characterized by clinical laboratory for the presence of autoimmune antibodies. Additionally, we ran a-dsDNA ELISA using TC-rich antigen SEQ1. For extended OUH cohort, commercial a-dsDNA ELISA kit was used as well (Supplementary Table S7). The results are shown in Supplementary Figure S1 and Table S7. As expected, 50–93% SLE samples showed elevated levels of a-dsDNA. In ELISA, RA and healthy controls showed weakly elevated a-dsDNA levels as well (10% RA (SSI), 10% healthy controls (SSI), 23% RA (OUH) and 28% healthy controls (OUH)). a-dsDNA positivity is not typical in RA^26^. Nevertheless 15–17% RA samples used herein were antinuclear antibody (ANA) positive, as evaluated by the commercially available ELISA test (Methods; Supplemental Figure S5, Table S7)^27^.

### DNA 50mer antigens: interaction with fluorophores and autoimmune antibodies

To target antibodies in patient sera, we initially designed three oligonucleotides as 50mer double-stranded helixes with different sequences^9, 28^ (SEQ1-3; Methods, Table 1). SEQ1, SEQ2 and SEQ3 were different in terms of nucleobase composition, containing TC, T + C and mixmer ATCG nucleotides, where +C is a locked nucleic acid (LNA) modification. Based on our recent report^9^, these compositions are favourable for specific interaction with SLE-related antibodies. However, to enhance the interaction with the dyes we extended the sequences to 50mers vs previously used 21mers.

**Table 1:** Synthetic dsDNA used in this study*.

Sequence, 5′-3′	Length (nt)
**SEQ1**: (TCC TCT CTT TCT CTT TCT CTT TCC TCT CTT TCT CTT TCT CTT TCC TCT CT):(AGA GAG GAA AGA GAA AGA GAA AGA GAG GAA AGA GAA AGA GAA AGA GAG GA)	50
**SEQ2**: (TCC + TCT CTT TCT + CTT TCT + CTT TCC + TCT CTT TCT + CTT TCT + CTT TCC + TCT CT):(AGA GAG GAA AGA GAA AGA GAA AGA GAG GAA AGA GAA AGA GAA AGA GAG GA)	50
**SEQ3**: (TGA ACT CTA TGT CTG TAT CAT TGA ACT CTA TGT CTG TAT CAT TGA ACT CT):(AGA GTT CAA TGA TAC AGA CAT AGA GTT CAA TGA TAC AGA CAT AGA GTT CA)	50
**SEQ4**: (TCC TCT CTT TCT CTT TCT CTT):(AAG AGA AAG AGA AAG AGA GGA)	21

^*^Pluses indicate LNA modifications.

**Figure 2:**
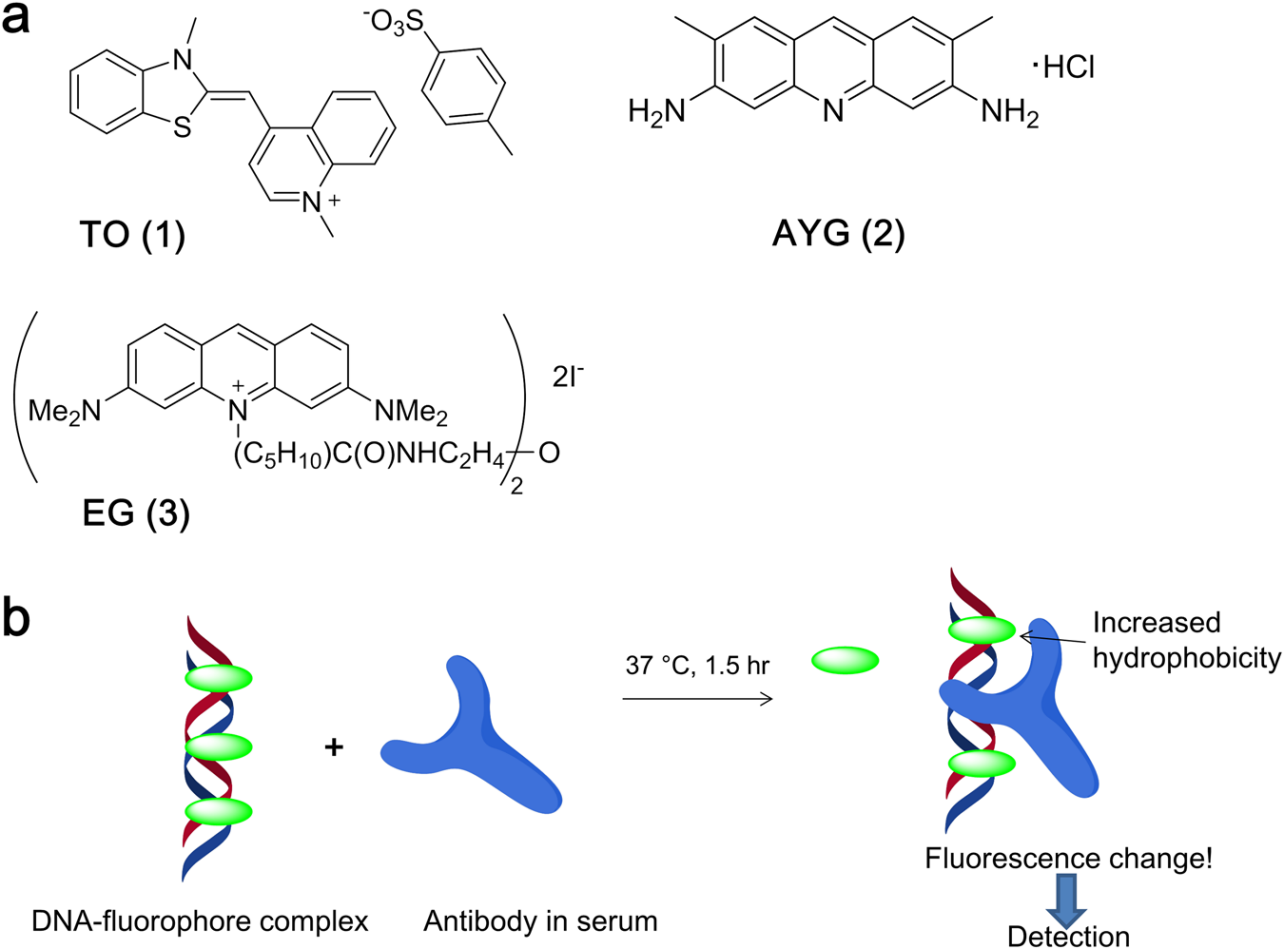
(**a**) Chemical structures of fluorescent dyes used in this work; (**b**) Main principle of the direct immunofluorescence assay developed herein.

Next, we selected three fluorescent dyes that are well known for interacting with dsDNA: intercalating dyes thiazole orange (TO) and acridine yellow G (AYG), and minor groove-binding Eva Green EG (Fig. 2a). We studied the interaction of these dyes with dsDNA 1–3 using fluorometry. Each dye was added to the mixture of two complementary DNA strands and the resulting mixtures were annealed (see methods). As expected, all the dyes showed quenched fluorescence in aqueous media and lit up upon binding to dsDNA (Supplementary Table S1). The brightest signal was observed for TO whereas the complexes of AYG and EG were 1.5–3-fold dimmer. Notably, the LNA rich sequence interacted less efficiently with the dyes and therefore was excluded from the study (Supplementary Table S1).

We applied SEQ1-dye complexes in an immunofluorescence assay (Fig. 2b). The rationale behind the assay was that the binding of an autoimmune antibody changes the number of dye molecules that are bound to DNA and also the micro-environmental parameters of the molecule. In particular, polarity and hydrophobicity are important for fluorescence intensity. We carried out the incubation of fluorescent DNA complex with control antibodies and patient sera for 1.5 hour at 37 °C, which is a physiologically the most relevant temperature^29^. Calf thymus DNA and single stranded synthetic DNA were used as controls^9, 28^. The result was detected by fluorometry as a change in the dye’s fluorescence intensity (Supplementary Figure S1). In the preliminary study of mono- and polyclonal antibodies SEQ1 and SEQ3 showed no binding of antibodies to β2-microglobulin, cardiolipin and HIV-1 protein associated antibody gp120 used as negative controls. Exclusively TO complex with LNA-modified SEQ2 showed a weak signal with anti-β2-microglobulin. On the contrary, calf thymus DNA (CTD) showed binding of all the negative controls, with the highest signal for anti-cardiolipin polyclonal antibodies. This was similar to the single-stranded controls SD1 and SD2 (Supplementary Table S2).

Next, using fluorescent complexes of SEQ1-3, we run the assay for eight SLE positive patient sera and 10 healthy controls. Based on the results (shown in Supplemental Figure S1), we made several important observations. First, no signal change was observed when DNA was not present. Next, there was a big difference in the response for each dye and DNA sequence. AYG showed low sensitivity to interaction with SLE sera, whereas EG and TO showed 1.7–3.4 fold fluorescence increase. Importantly, we observed no signal change for EG and TO complexes with SEQ1-2 in the healthy controls. However, the signal change upon incubation was below the detection limit needed for diagnostic applications (3-fold above background of the microtiter plate). We conclude that the number of dyes interacting with antigens had to be increased in order to improve the sensitivity of antibody detection.

### DNA origami as antigens for immunofluorescence assay

In the next step we hypothesized that 3D DNA nanostructures with their extended size and well-defined shape could be favourable for the binding of multiple fluorescent dyes and also for an effective interaction with antibodies in patient sera^30, 31^. To check this hypothesis we selected two DNA origami structures: a 100 nm × 70 nm 2D rectangle (TR) and six-helix bundle (6HB) rod approximately 400 nm in length. To prepare the origami structures and introduce fluorophores we used a thermal annealing protocol (methods). EG, TO and AYG were added in different concentrations at 60 °C. This was done in order to prevent dye degradation and ensure its successful incorporation into the nanostructure^32^.

We studied the obtained fluorescent origami complexes by fluorometry (Supplementary Figure S2). A striking finding was that TO did not show any difference in fluorescence upon incubation with origami, even when the protocol was changed and TO was added at 90 °C. On the contrary, EG showed the highest fluorescence with both structures, with a remarkable signal-to-background ratio of 30. Therefore we chose origami-EG complexes for further immunofluorescence assay. AYG was excluded due to low sensitivity to interaction with human sera mentioned above.

The dye concentration in the annealing mixture also had an interesting effect on the fluorescence properties of the complex. Approximately 0.5X EG solutions gave extremely bright fluorescent complexes. However, using more EG dye (1-2X), the fluorescence of the resulting nanostructure complexes was quenched 2-fold and more compared to the complexes with a more diluted dye (Supplemental Figure S3).

**Figure 3:**
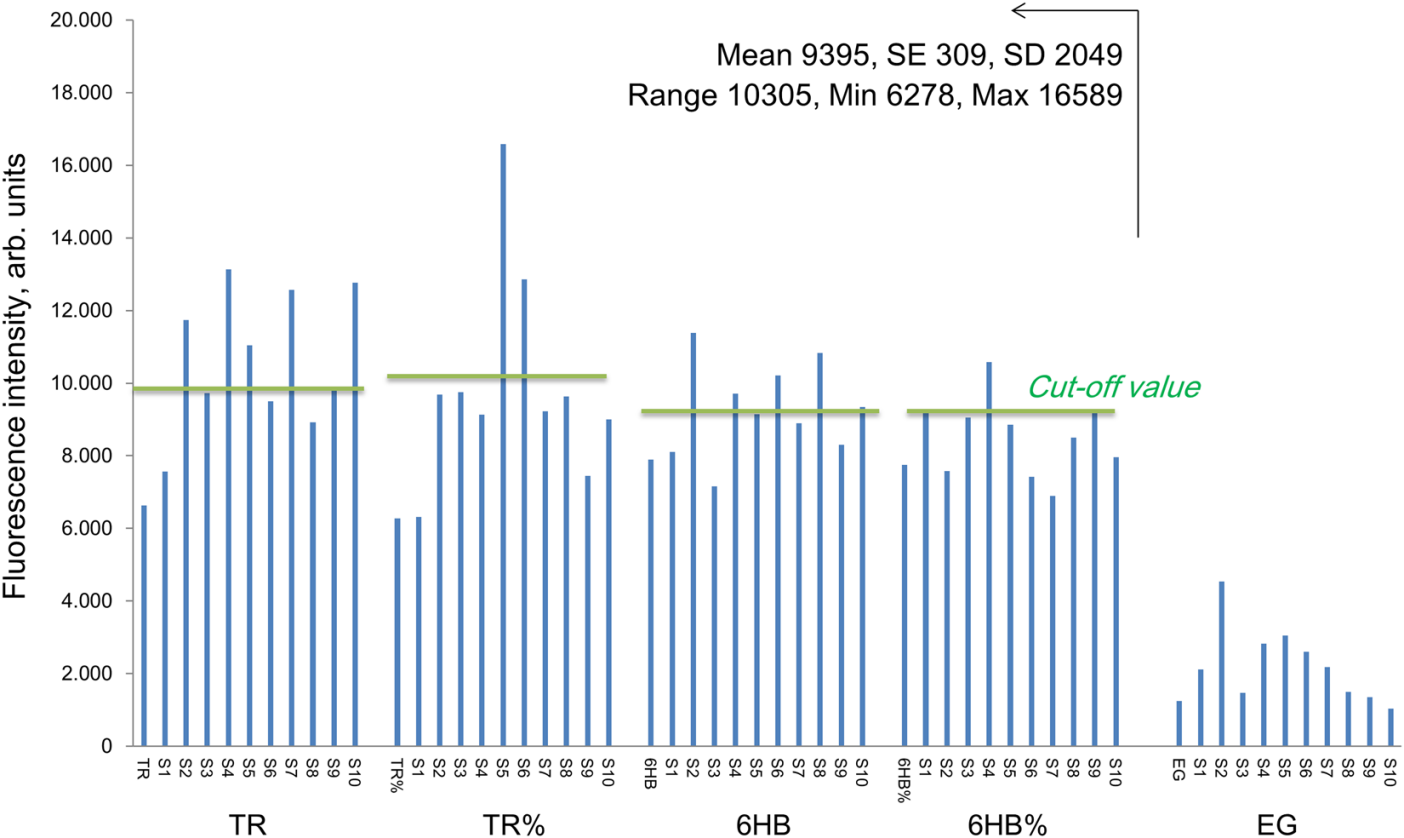
Immunofluorescence assay results using DNA origami and Eva Green and SLE sera. Cut-off values are shown as green lines; they were determined for each antigen as 2 standard deviations above the mean value for response in a non-matched healthy control cohort (n = 20). TR, 6HB = assembled DNA origami; TR%, 6HB% = origami sequences without bacteriophage DNA. S1–S10 = SLE patient samples. EG = Eva Green.

**Figure 4:**
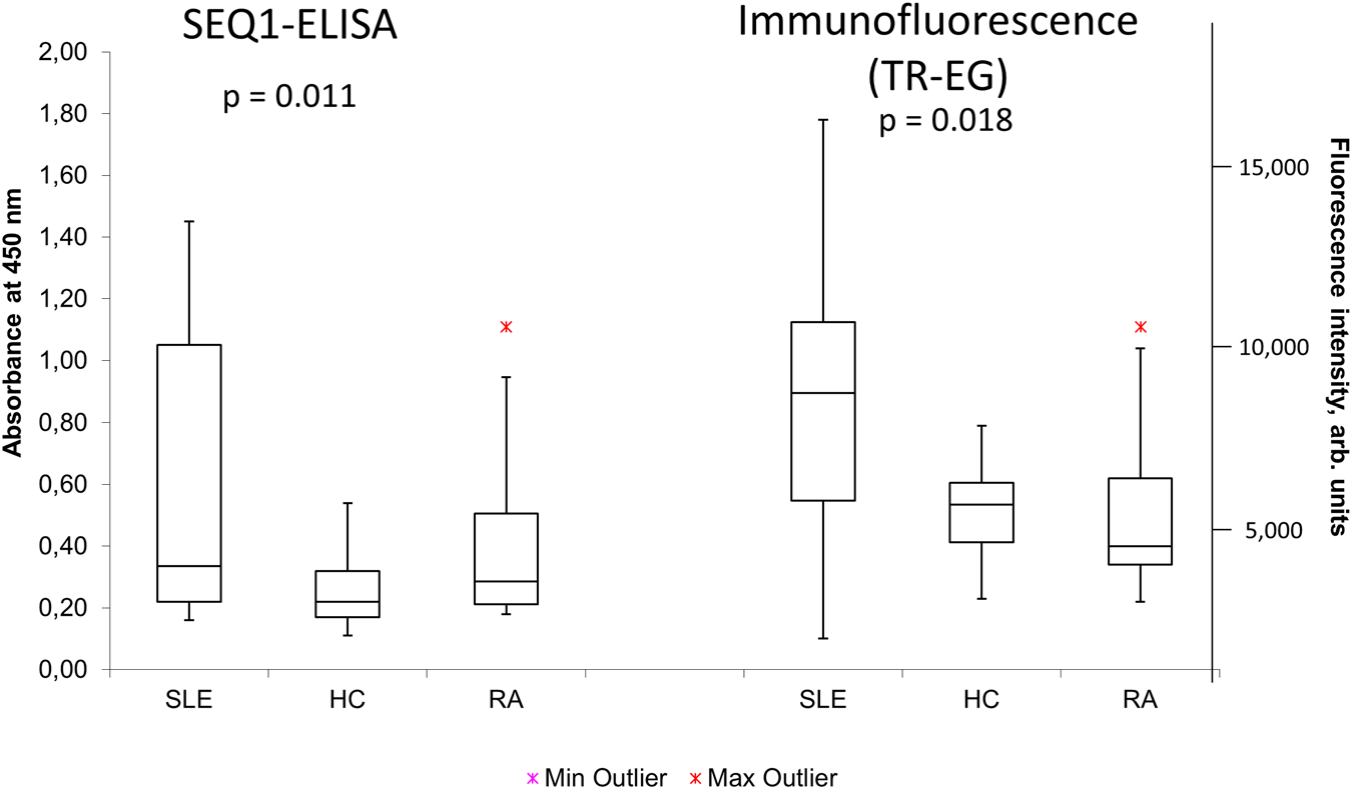
Box-and-whisker plot with outliers for the results of ELISA and immunofluorescence assays performed for SSI samples: SLE (n = 28), HC (n = 20) and RA (n = 20). The arms on each boxplot are values Q_1_–1.5 × IQR and Q_3_ + 1.5 × IQR. Data points for each subject are means for three independent measurements. TR-EG = TR origami complex with Eva Green dye. Standard error bars for independent triplicate experiments (n = 3) across antigens are shown in Supplementary Figure S4.

For immunofluorescence, complexes of TR and 6HB with EG dye in the optimal dilution regimen were incubated with SLE sera and controls as described for 50mer duplexes (Supplemental Tables S3–S7). The resulting fluorescence intensity is shown in Figs 3 and 4. First, the background signal of Eva Green dye in the absence of origami did not differ from the background of empty microtiter plates (Fig. 3, EG group). Next, 50% SLE (SSI) and 93% SLE (OUH) had a positive signal upon incubation with the TR-EG complex (Fig. 4; Supplemental Tables S6, S7). Notably, only a few samples showed a positive signal when origami controls without the large bacteriophage DNA were applied. Furthermore, we also observed a difference in signal between the TR-EG and 6HB-EG complexes. The difference in fluorescence intensity for all tested groups was statistically significant with p = 0.018 as confirmed by one-way ANOVA analysis in R (Methods)^33^. Lastly, using a different origami structure or excluding the bacteriophage sequence from it lead to statistically significant difference in response when the same patient samples were used (p = 0.07 and 0.04 for TR‒6HB and TR‒TR% groups).

Using healthy cohort, we additionally compared performance of TR-EG to calf thymus DNA-EG (Supplementary Table S2). In a good agreement with our recent study^9^, CTD showed a high number of false positive results in healthy controls (31%), which points on non-specific interactions.

Sensitivity and specificity of the developed assay to SLE were studied using additional patient samples obtained from OUH, Denmark (Supplementary Table S7). Being compared to the commercial a-dsDNA ELISA test, TR-EG immunofluorescence method showed superior specificity to SLE (94% vs. 81%), whereas the sensitivity was similar for both assays (93%). In turn, when compared to SEQ1-ELISA, the specificity was similar, although the former method had slightly lower sensitivity to SLE (80% vs. 93%). Remarkably, the developed immunofluorescence assay showed lower number of positives in healthy controls when compared to ELISA (3% vs. 13% and 10% for immunofluorescence vs. commercial and in-house SEQ1-ELISA; n = 60). Number of positives in other rheumatic diseases (RA, APS) was also lower for IFA and SEQ1-ELISA than for the commercial a-dsDNA test (7–8% vs. 18%; Supplementary Table S7). In Crohn’s disease, hepatitis B and cancer, the performance of TR-EG assay was slightly better than for SEQ1-ELISA (14% vs. 9%. positives), and superior to the commercial ELISA which gave 32% positives in these samples.

**Figure 5:**
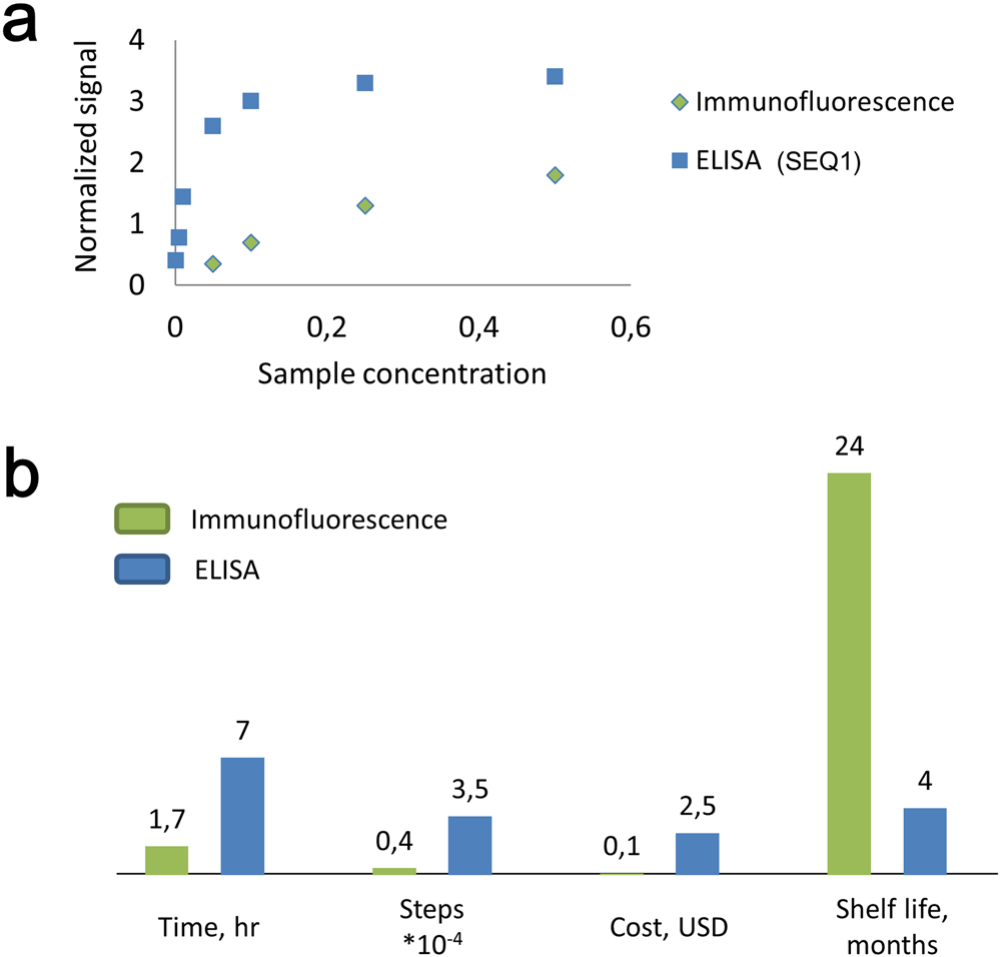
Comparison of SEQ1-ELISA and immunofluorescence assays: (**a**) limit of target detection determination. Signal at each point is a mean value for triplicate measurement of randomly selected 5 SLE samples; (**b**) (*left to right*) time, step count per one assay; cost per sample analysed, shelf-life time of antigen. Each measurement has been done three times with a result deviation ±3%.

As a final aspect, we explored the limitations and benefits of each assay format by a comparative study of the limit of target detection (LOD), cost, labour demand and speed (Fig. 5; see methods for details). The developed immunofluorescence using TR-EG complex had 10-times higher LOD than SEQ1-ELISA. However, immunofluorescence was much faster, less expensive and less labour-intensive. This is accompanied by a higher stability of the origami reagents applied for immunofluorescence when compared to precoated ELISA plates (Fig. 5c). ROC curves are often used to compare specificity and sensitivity of the assays at different cut off values^34^. In agreement with close LOD values, receiver-operating characteristic (ROC) curves were rather similar for ELISA and immunofluorescence assays (Supplemental Figure S6; Supplemental Table S8).

## Discussion

The purpose of this work was to investigate the possibility of using sequence-controlled short DNA antigens and well-defined 3D DNA structures as new reagents that may have the potential to discriminate among anti-DNA antibodies and advance existing detection methods. As we demonstrate in this paper, using DNA origami and the minor groove-binding dye Eva Green, a fast immunofluorescence assay can be performed for the detection of antibodies towards dsDNA. The method takes advantage of the fact that the number of dyes interacting with DNA changes when the antibody interacts with the DNA-dye complex, Since fluorescence of the dye is quenched in aqueous media and increases in hydrophobic environments, such as the DNA groove or antibody epitope, the interaction between the dye-DNA and antibody can be traced by the fluorescent signal of the dye.

This work results in two major findings. First, the 3D structure of DNA has an impact on the interaction with disease-specific autoimmune antibodies. In particular, our studies confirm that the well-defined structure of DNA antigen is beneficial for the interaction with a-dsDNA of preferably the IgG type. This is also proved by the negative result of our control experiments when a simple mixture of origami primers is used as antigen. Second, the binding of fluorophores to large mixmer DNA origami ensures the generation of enough signal when a high-affinity antibody ‘removes’ the dye and interacts with the DNA antigen. This could not be achieved when using shorter dsDNA since not enough dye molecules were bound and therefore the signal upon antibody recognition was too weak. Thus, multiple interaction sites for the dye on the DNA origami act as a signal ‘booster’ for the assay, and hence allow a simple, single-step immunofluorescence homogeneous method to be carried out.

The interaction of diverse DNA antigens with fluorophores and consequent binding of polyclonal antibody targets by the DNA-dye complexes are fundamental interactions that we investigate in this work. An interesting finding is that intercalating and groove-binding dyes have different binding properties for short dsDNA and large nanostructures such as DNA origami. Short dsDNAs effectively bind the DNA intercalator thiazole orange and show less binding with groove-binding Eva Green. For DNA origami the situation is just the opposite: thiazole orange shows no interaction whereas Eva Green generates stable, bright complexes.

This work lies in the context of recent research on fluorescent DNA-protein interactions, although the reports on 3D DNA-protein interactions are limited, especially as tools for the diagnostics of autoimmune diseases. Recently, DNA nanostructures were proposed for the directed recognition of proteins^35^. The interaction of origami with antibodies has also been demonstrated before, although not in the context of diagnostics and the study of autoimmune diseases but for the detection of origami via ligand-specific aptamer sequence^36^.

Our assay shows that the structure of origami has an influence on the antibody binding. Thus, TR shows a bright positive signal and a statistically significant difference upon interaction with antibodies in SLE- and RA-positive sera, whereas for 6HB the overall signal is lower and there is no statistically significant difference in the signal for patient samples vs. controls. Once again, this implies that in addition to sequence specificity, the 3D structure of DNA antigen might have an influence on its recognition by the autoimmune antibodies^37–39^. This has been previously observed for small DNA, however the detailed structure of these DNA-antibody complexes has not been studied^40^. This could be due to the fact that NMR and X-ray studies of DNA complexes are experimentally challenging^41^. This makes us believe that this new immunofluorescence assay has a high potential as a tool to gain new valuable knowledge on DNA-protein complexes, and in particular on clinically relevant autoimmune antibodies, as demonstrated in this paper.

Generally, the immunofluorescence assay proposed herein has comparable sensitivity and specificity to SLE as ELISA (81–93% and 80–94%), but 10-fold higher LOD. Nevertheless, a rather small amount of patient sera (2 µL per analysis) was sufficient for the detection of a-DNAs in SLE-positive sera. Notably, the developed method shows 10–15% of positives in RA, whereas ELISA gives 10–23% positives in RA. RA and SLE share similar autoimmune features. Therefore it is expected that there is some overlap in the biomarker profiles of these diseases^16–18^. Additional tests needed to distinguish RA from SLE include complement components (C3, C4), erythrocyte sedimentation rate, cyclic citrullinated peptide antibody and rheumatoid factor^42, 43^.

It is advantageous that the length of the assay is reduced from 6.5 hr for indirect ELISA to only 1.5 hr, which is comparable to direct immunofluorescence assay (DIF)^44^. However, the cost of new immunofluorescence assay per sample is much lower than for ELISA and DIF since it does not require the use of expensive antibody reagents. Moreover, the origami reagent has a much longer shelf life (2 years at −20 °C and >5 years at −80 °C, vs. 4 month for pre-coated ELISA plates at +4 °C; see methods). Immunofluorescence is also way less labour-intensive and therefore could become a useful tool in the diagnostics of clinically and biologically relevant antibodies.

In conclusion, we show that disease-specific autoimmune antibodies can discriminate the 3D structure of macromolecular DNA antigens. We confirm improved specificity of synthetic double-stranded complexes compared to currently applied CTD and single stranded DNA. We also demonstrate that non-covalent complexes, such as DNA origami with groove-binding dye, can be a useful tool in fast and specific detection of autoimmune antibodies. The biological role of 3D DNA structure in interactions with autoantibodies remains to be fully elucidated. In this study, we have taken an initial step to facilitate this exploration and to provide a new tool that merits further exploration for assessment of DNA-binding antibodies in autoimmunity with potential for differentiating among distinct diseases.

## Methods

### General

Oligonucleotides SEQ1-3 (Table 1) were synthesised in-house using the phosphoramidite method^45^. All other nucleic acid compounds were obtained from Integrated DNA technologies, Inc., Iowa, USA. Fluorescence dyes were purchased from Sigma (AYG, TO) and Biotium (EG) and used as received. Calf thymus DNA (CTD) was purchased from Sigma (cat no. D1501).

ELISA assays were made manually using following the protocol described recently^9^. Commercial a-dsDNA ELISA kit was purchased from Abcam (cat. no. ab178618). Plates were analysed using a TECAN microplate reader and measuring absorbance at 450 nm. 96-well Maxisorb NUNC microplates were purchased from Thermofisher Scientific.

Patient sera samples and healthy controls were obtained from Odense University Hospital and Statens Serum Institute, Denmark. Monoclonal antibodies were provided by Statens Serum Institute, Denmark (anti-dsDNA and anti-β2-microglobulin), purchased from commercial supplier (anti-cardiolipin, Immunovision, cat no HCL-0200; gp120 (Abcam, cat no ab21179). Written approval by The Danish Data Protection Agency was obtained in November 2015. Committee of Information Safety of the Region Southern Denmark at the Danish Data Protection Agency specifically approved the whole study (permission signed by Pernille Winther Christensen). The methods were carried out in accordance with the relevant guidelines and regulations as stated in the Act on Processing of Personal Data adopted by the Danish Data Protection Agency on June 2^nd^ 2000. Personal data of patients was not used in this work. Therefore the informed consent from the individuals was not needed.

Price per assay has been estimated using costs for oligonucleotides, dyes, other reagents and equipment provided by commercial suppliers (IDT, Sigma).

Step count is determined using S health application (Samsung galaxy S5) as an average number of steps for an individual while performing the assay.

Antigen stability upon storage was determined by MALDI-MS and ELISA for synthetic ODNs and precoated plates, respectively.

### 50mer DNA Antigens and 21mer ELISA control

Sequences SEQ1-4 used in this study are shown in Table 1.

Origami sequences were designed and prepared following published procedures^46, 47^. Annealing procedure for origami and controls was carried out using 10 nM samples in 1x TAE buffer with 12 mM MgCl_2_ as follows: lid 100 °C, 90 °C 2 min, 90–60 decrease 0, 5 °C pr 1 min, 60–50 decrease 0, 2 °C pr 10 min, 50–35 decrease 0, 5 °C pr 1 min; store at RT or 10 °C.

**ANA** kit was purchased from Abcam (product no. ab178610) and used following manufacturer’s protocol.

### General protocol for immunofluorescence assay

In a microplate, DNA-dye antigen (4 μL, 10 nM) was mixed with 4 μL freshly prepared diluent (1 g BSA, 200 μL Tween-20 in 1 L 1 × PBS). Afterwards 2 μL serum sample was added. Incubation was performed for 1.5 h at 37 °C, followed by immediate fluorescence detection at Light Cycler 480 reader (emission at 530 nm). Incubation time and volume were optimized by a series of experiments carried out for 30 min–2 hrs, using 2–50 μL incubaton reactions (see Supplemental information).

**Data analysis** was performed in R using one-way ANOVA^33^.

## Electronic supplementary material

Supplementary Information
