# Supplementary material for: Complexes of DNA with fluorescent dyes are effective reagents for detection of autoimmune antibodies
Source: Sci Rep. 2017 May 15;7:1925. doi: 10.1038/s41598-017-02214-0 (PMC5432514; doi:10.1038/s41598-017-02214-0)
Supplement: Supplementary file 1 — Supplementary Information [file 41598_2017_2214_MOESM1_ESM.pdf]

Supplemental information for the paper:

Complexes of DNA with fluorescent dyes are effective reagents for detection of autoimmune antibodies

Ivana Domljanovic,[1] Annika Carstens,[1] Anders Okholm,[2] Jørgen Kjems,[2] Christoffer Tandrup Nielsen,[3] Niels H. H. Heegaard[4,5] and Kira Astakhova[1,*]

* Corresponding author: ias@sdu.dk

Table of contents

**List of abbreviations**	S2
**I. Pilot studies of DNA-dye complexes**	S3
**Supplemental Table S1.** Fluorescence intensities of DNA-fluorophore complexes	S3
**Supplemental Table S2. Analysis of binding specificity for DNA-dye antigens and controls**	S3
**Supplemental Figure S1.** Immunofluorescence assay results for SEQ1 with TO and EG upon incubation with human samples	S5
**II. Immunofluorescence assay using TR-EG complex – Assay development**	S6
**Supplemental Figure S2.** Fluorescence intensities for DNA origami complexes with fluorophores and controls.	S6
**Supplemental Tables S3-S5.** Immunofluorescence assay using TR origami and SLE sera at different concentrations.	S7
**Supplemental Figure S4.** Standard error bars for independent triplicate measurements of SLE samples using TR-EG complex.	S12
**III. Comparison of immunofluorescence with ELISA**	S13
**Supplemental Table S6. Results for individual patients used in this study**	S16
**Supplemental Table S7. Sensitivity and specificity assessment of the extended patient cohort (OUH)**	S17

**Supplemental Table S7. ROC Table for ELISA and immunofluorescence assays**	S18
**Supplemental Figure S6. ROC curves for ELISA and TR-EG immunofluorescence assay**	S19

List of abbreviations

Anti-DNA – antibody to DNA

ELISA – enzyme-linked immunosorbent assay

DIF – direct immunofluorescence assay

IIF - indirect immunofluorescence assay

IFA – immunofluorescence assay

SPR – surface plasmon resonance

SLE – systemic lupus erythematosus

RA – rheumatoid arthritis

APS – antiphospholipid syndrome

HC – healthy control

EG – Eva Green

AYG – acridine yellow G

TO – thiazole orange

TR – Tall rectangle

6HB – Six-helix bundle

I. Pilot studies of DNA-dye complexes

Supplemental Table S1. Fluorescence intensities of DNA-fluorophore complexes*

SEQ#	Dye: EG	TO	AYG
**1**	1210	4450	1120
**2**	930	2890	765
**3**	1100	3320	1300
**4**	320	980	260
**none**	120	440	200

* Each value is a mean value of three independent measurements with the result deviation ±3%. Excitation wavelength = 500 nm, emission wavelength = 530 nm.

Supplemental Table S2. Analysis of binding specificity for DNA-dye complexes and control antibodies.[a]

Antigen No/details	Binding human monoclonal antibodies:				a-CL polyclonal antibodies	Response in healthy controls, Odense University Hospital, HNP (% of patients)
	a-dsDNA	a-β2m	gp120			
**SEQ1-EG**	+	-	-		-	+/- (6%)
**SEQ2-EG**	+	-	-		-	+ (6%), +/- (6%)
**SEQ3-EG**	+	-	-		-	-
**SD1-EG**	+/-	-	+/-		+	+/- (31%)
**SD2-EG**	+/-	-	+/-		+	+/- (31%)
**CTD-EG**	+	+/-	+/-		+	+ (31%), +/- (31%)
**EG**	-	-	-	-	-	-
**SEQ1-TO**	+	-	-		-	+/- (6%)
**SEQ2-TO**	+	-/+	-		-	+/- (6%)
**SEQ3-TO**	+	-	-		-	+/- (6%)

**SD1-TO**	+/-	-	+/-		+	+/- (50%)
**SD2-TO**	+/-	-	+/-		+	+/- (44%)
**CTD-TO**	+	+/-	+/-		+	+ (44%), +/- (31%)
**TO**	-	-	-	-	-	-

[a] Weak positive (+/-) and positive (+) signal were defined as 2- and 3-fold absorbance signals, respectively, above the mean value for a healthy control group. SD1 and SD2 are single stranded DNA: SD1, 5’-TCC TCT CTT TCT CTT TCT CTT TCC TCT CTT TCT CTT TCT CTT TCC TCT CT-3’; SD2, 5’-TGA ACT CTA TGT CTG TAT CAT TGA ACT CTA TGT CTG TAT CAT TGA ACT CT-3’.

CTD = calf thymus DNA. CL = cardiolipin.

Antibodies were chosen based on our previous reports (Samuelsen et al. PLOS One 2016, Sci Rep 2016).

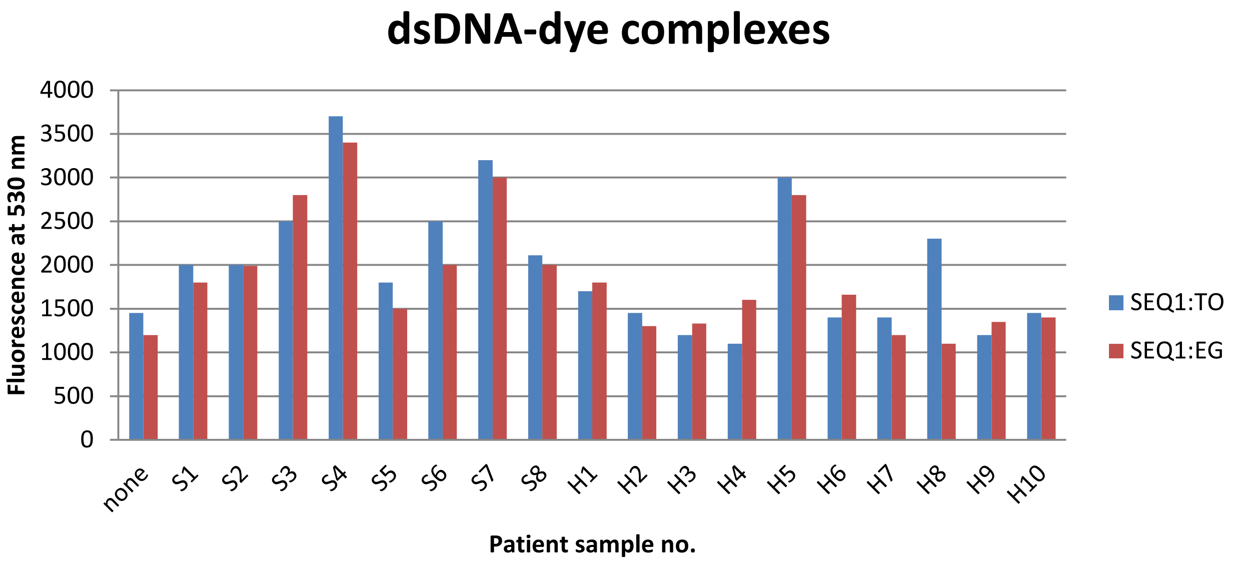

Supplemental Figure S1. Immunofluorescence assay results for SEQ1 with TO and EG upon incubation with human samples in dilution 1:5 (SSI cohort). Signals in the absence of sera: SEQ1:TO, 4450; SEQ1:EG, 1210.

II. Immunofluorescence assay using TR-EG complex – Assay development

To develop the assay, we considered the following: sample volume, amount of the dye/DNA reagent, serum concentration, and incubation time. These parameters have been varied for the assay using five randomly selected SLE samples and five healthy controls (SSI cohort). Based on this study, optimal signal to background ratio at highest signal was observed for 10 µl total sample volume using 2 µl sera and 4 µl 10 nM dye-origami reagent (Tables S1-S3). Optimal incubation time is 1.5 hr (Figure S4).

a)

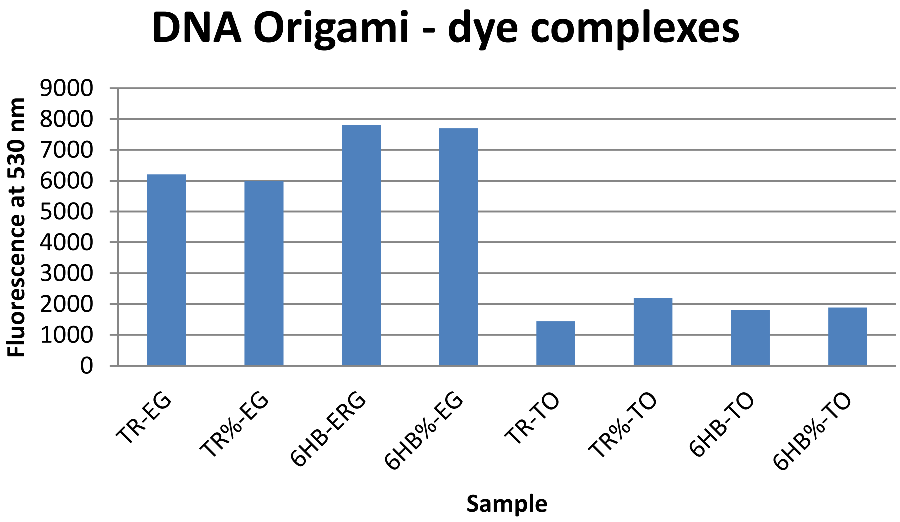

b)

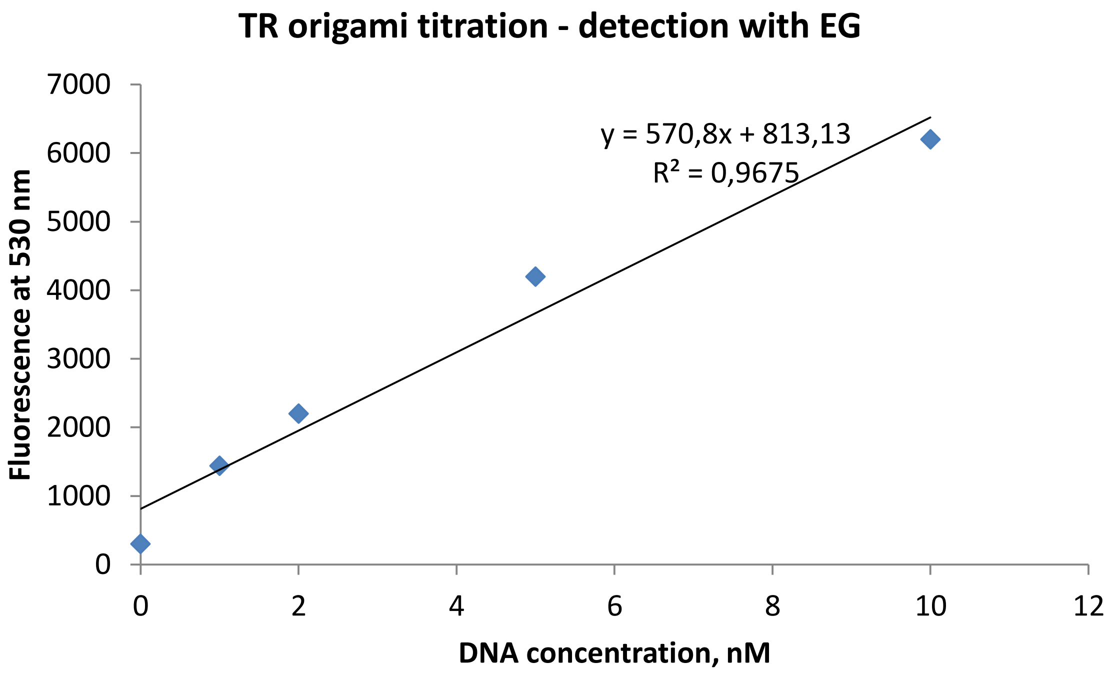

c)

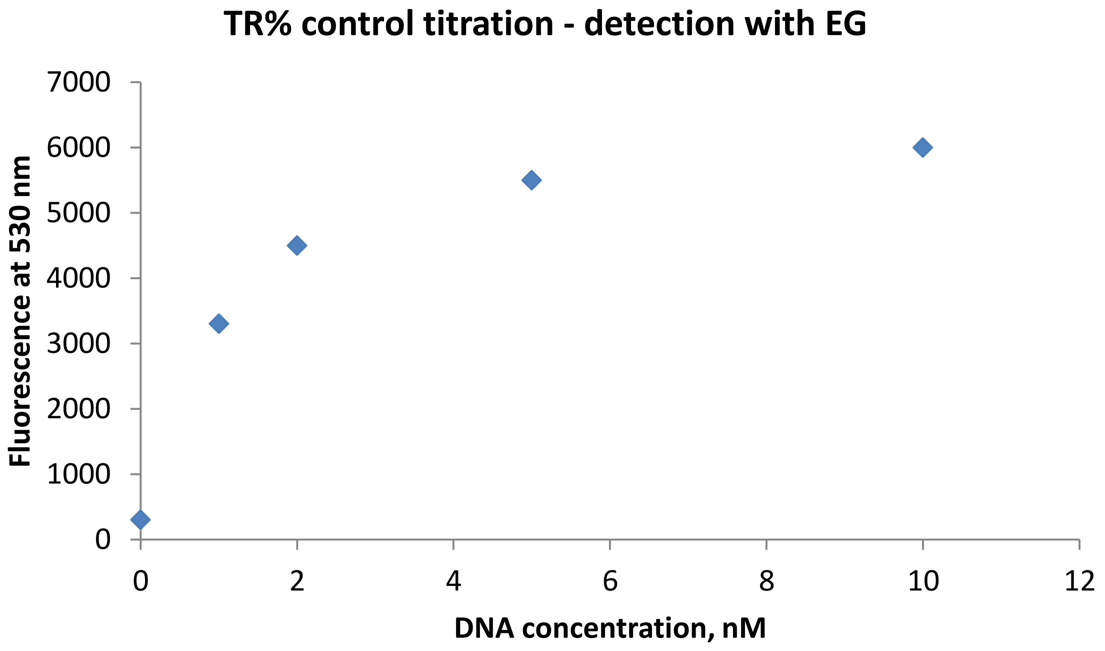

Supplemental Figure S2. Fluorescence intensities for DNA origami complexes with fluorophores and controls, measured in 1xPBS buffer (pH 7.2), at 37 °C. DNA concentrations were 10 nM (a), and serially diluted to 2 nM (b-c). Excitation/emission wavelengths were 500 nm/530 nm.

Supplemental Table S3. Immunofluorescence assay using TR origami and SLE sera at different concentrations (SSI cohort).

TR concentration: 4 nM

Total V, µL	Sera V, µL	Signal	Signal to BG ratio
10	0	6200	
10	0,5	6900	1,113
10	1	6300	1,016
10	1,5	9000	1,452
**10**	**2**	**14300**	**2,306**
10	3	12100	1,952
20	0	3300	
20	0,5	3300	1,000
20	1	3900	1,182
20	1,5	4700	1,424
20	2	7700	2,333
20	3	6200	1,879
30	0	2100	
30	0,5	2000	0,952
30	1	2100	1,000
30	1,5	2500	1,190
30	2	3400	1,619
30	3	5000	2,381
50	0	980	
50	0,5	990	1,010
50	1	1000	1,020
50	1,5	1010	1,031
50	2	1400	1,429
50	3	1600	1,633

Each data point is an average signal for 5 randomly selected SLE samples. Used EG dye: 10 µL 20X stock in 100 µL DNA sample.

Supplemental Table S4. Immunofluorescence assay using TR origami and SLE sera at different concentrations (SSI cohort).

TR concentration: 2 nM			
Total V, µL	Sera V, mkl	Signal	Signal to BG ratio
10	0	2920	
10	0,5	3300	1,130
10	1	2700	0,925
10	1,5	4200	1,438
10	2	6600	2,260
10	3	4500	1,541
20	0	1450	
20	0,5	1440	0,993
20	1	1700	1,172
20	1,5	2200	1,517
20	2	3560	2,455
20	3	2700	1,862
30	0	900	
30	0,5	940	1,044
30	1	980	1,089
30	1,5	1120	1,244
30	2	1600	1,778
30	3	2300	2,556
50	0	440	
50	0,5	470	1,068
50	1	480	1,091
50	1,5	465	1,057
50	2	650	1,477
50	3	900	2,045

Each data point is an average signal for 5 randomly selected SLE samples. Used EG dye: 10 µL 20X stock in 100 µL DNA sample.

Supplemental Table S5. Immunofluorescence assay using TR origami and SLE sera at different concentrations (SSI cohort).

**TR concentration: 8 nM**			
Total V, µL	Sera V, µl	Signal	Signal to BG ratio
10	0	10100	
10	0,5	12300	1,218
10	1	14500	1,436
10	1,5	19000	1,881
10	2	14300	1,416
10	3	11200	1,109
20	0	5400	
20	0,5	7000	1,296
20	1	11000	2,037
20	1,5	12400	2,296
20	2	12900	2,389
20	3	11340	2,100
30	0	3400	
30	0,5	3550	1,000
30	1	5400	1,044
30	1,5	8600	1,588
30	2	9900	2,529
30	3	10300	2,912
50	0	2300	
50	0,5	1910	1,000
50	1	2550	0,830
50	1,5	3600	1,109
50	2	5400	1,565
50	3	7000	2,348

Each data point is an average signal for 5 randomly selected SLE samples. Used EG dye: 10 µL 20X stock in 100 µL DNA sample.

a)

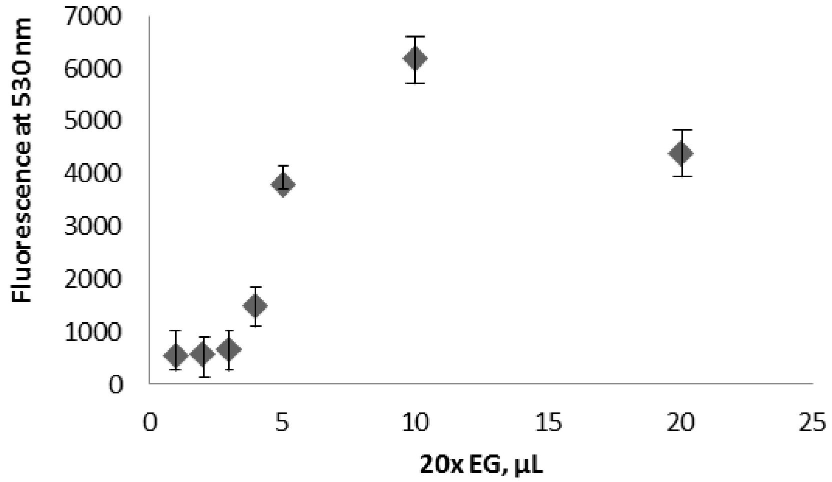

b)

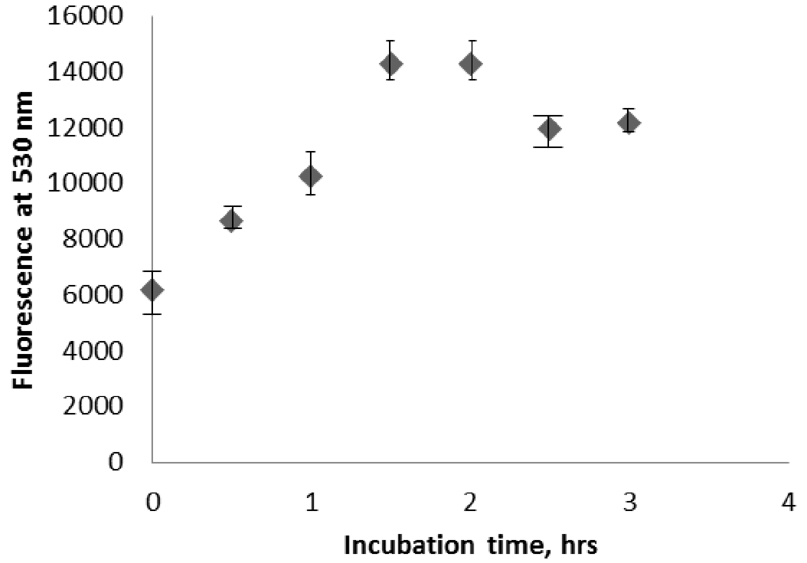

Supplemental Figure S3. Results for immunofluorescence assay at different conditions (SSI cohort). a) 10 nM DNA origami in a total volume 100 mkl 1xPBS, pH 7.2, was used. Every data point is an average of three independent measurements with deviation in the result below 3%. For b), DNA-DNA complex was prepared by annealing 10 mkl EG 20x stock in total 100 mkl sample using 10 nM TR DNA origami. This complex was then added to 2 mkl sera in total 10 mkl incubation buffer as described above.

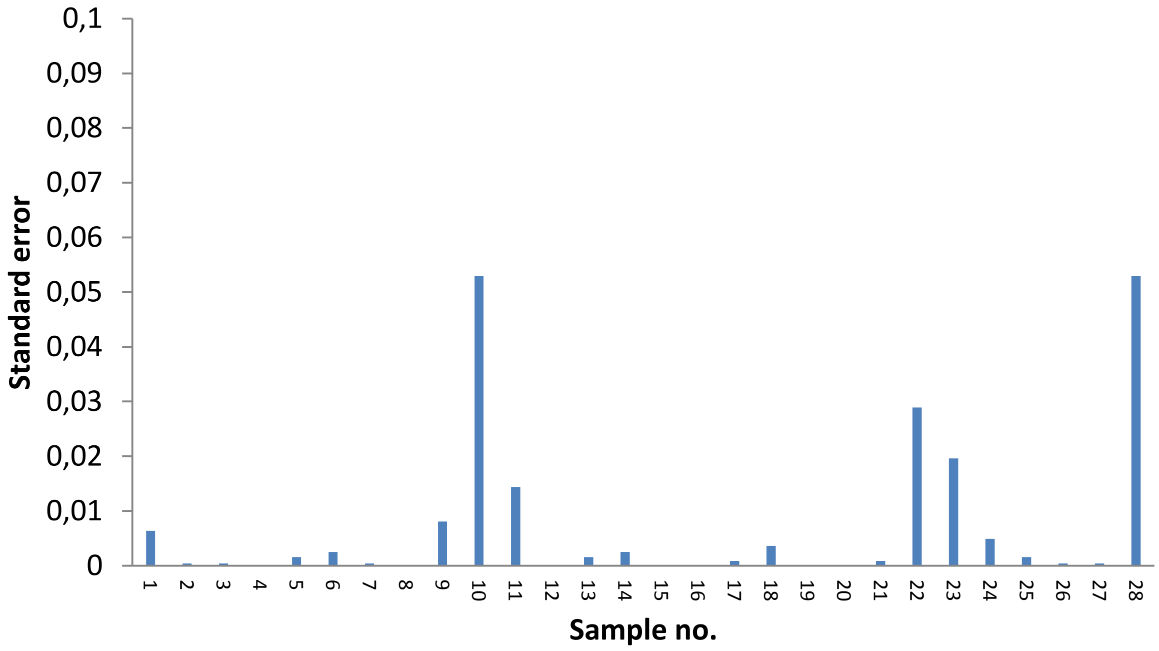

Supplemental Figure S4. Standard error bars for independent triplicate measurements of SLE samples using TR-EG complex (SSI cohort).

III. Comparison of immunofluorescence assay with ELISA – SSI cohort

a)

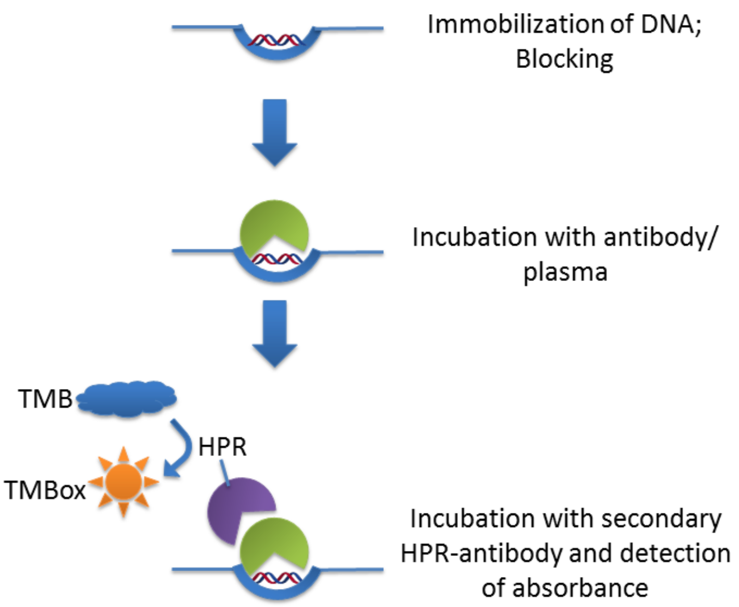

b)

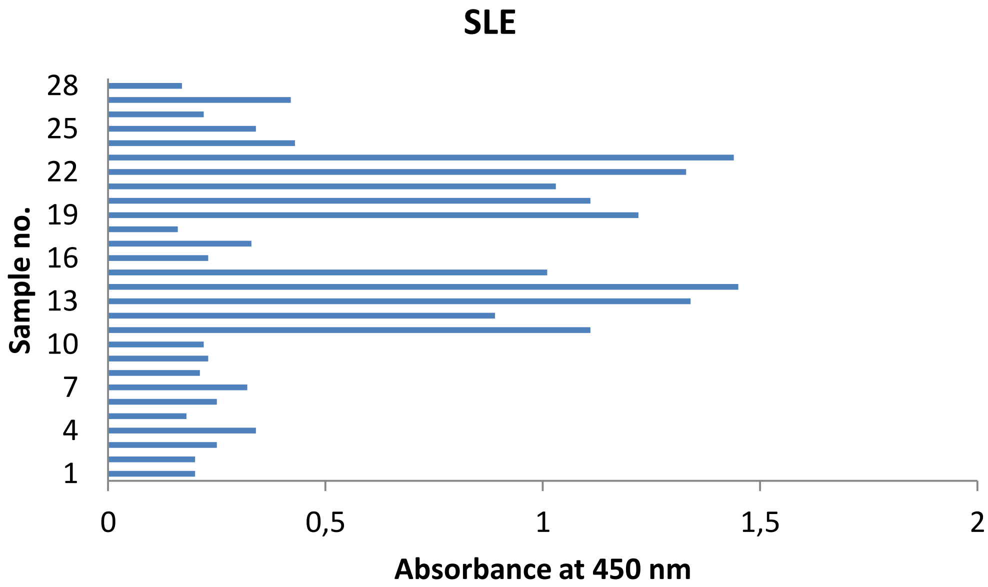

c)

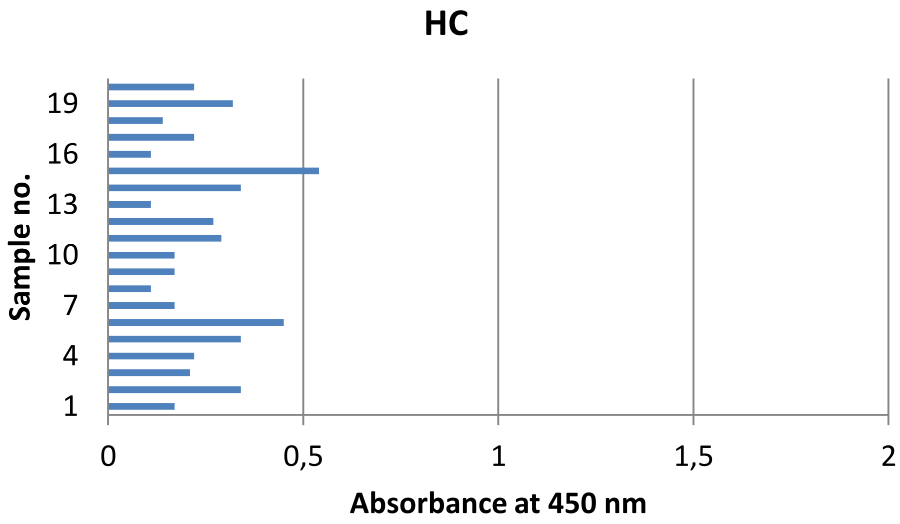
HC
Sample no.
19
16
13
10
7
4
1
0
0,5
1
1,5
2
Absorbance at 450 nm

d)

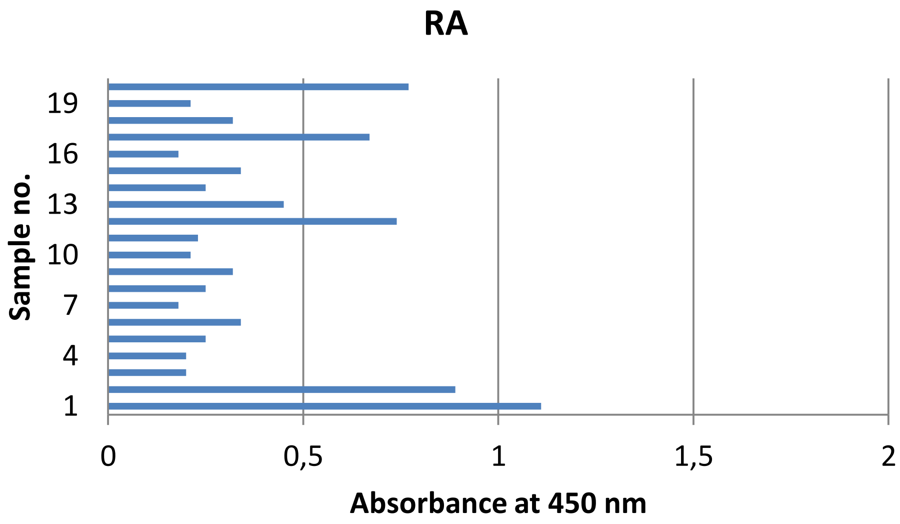
RA
Sample no.
19
16
13
10
7
4
1
0
0,5
1
1,5
2
Absorbance at 450 nm

e)

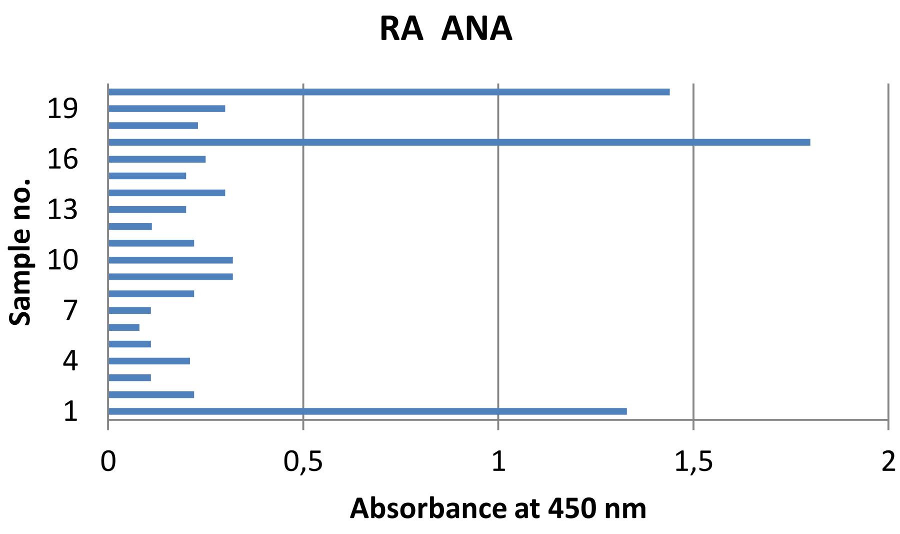

Supplemental Figure S5. General scheme of indirect ELISA assay (A) and results (B-E) for patient samples, SSI cohort.

Supplemental Table S6. Results for individual patients used in this study (SSI cohort).*

	ELISA	IFA		ELISA	IFA		ELISA	IFA
Pat.no.	SLE	SLE	*Pat.no.*	HC	HC	*Pat.no.*	RA	RA
1	0	0	*1*	0	0	*1*	3	1
2	0	0	*2*	0	0	*2*	2	1
3	0	1	*3*	0	0	*3*	0	0
4	1	1	*4*	0	0	*4*	0	0
5	1	1	*5*	0	0	*5*	0	0
6	1	1	*6*	1	0	*6*	0	0
7	0	0	*7*	0	0	*7*	0	0
8	0	0	*8*	0	0	*8*	0	0
9	0	0	*9*	0	0	*9*	0	0
10	0	0	*10*	0	0	*10*	0	0
11	3	1	*11*	0	0	*11*	0	0
12	3	2	*12*	0	0	*12*	0	0
13	3	2	*13*	0	0	*13*	0	0
14	3	1	*14*	0	0	*14*	0	0
15	3	1	*15*	1	0	*15*	0	0
16	0	0	*16*	0	0	*16*	0	0
17	0	0	*17*	0	0	*17*	0	0
18	0	0	*18*	0	0	*18*	0	0
19	3	2	*19*	0	0	*19*	0	1
20	3	2	*20*	0	0	*20*	0	0
21	3	2						
22	3	2						
23	3	2						
24	0	0						
25	0	0						
26	0	0						
27	0	0						
28	0	0						

* Cut off values were calculated as 2xstandard deviations (SD) above healthy control cohort response for weakly elevated (1), 3X SD and 4xSD for positive (2) and highly positive samples (3), respectively.[1] Cut-off values for ELISA, absorbance at 450 nm: > 0.66 (1), > 0.88 (2) and > 1.10 (3); Values for IFA, fluorescence at 530 nm, * 10^4: > 0.5 (1), > 1.0 (2) and > 1.5 (3).

[1] Samuelsen et al. PLOS One 2016

Supplemental Table S7. Assay comparison using extended disease stated sera samples and healthy controls (OUH cohort).*

	Measured samples	Abcam ELISA positives	SEQ1-ELISA SDU, positives	IFA positives
SLE, ANA+	28	26	24	26
Crohn's disease	10	4	2	1
RA	30	**7**	2	**4**
APS	30	5	2	1
Hepatitis B	4	1	0	0
Cancer	8	2	1	1
Healthy controls	60	**8**	**6**	**2**
Sensitivity to SLE, %		93	80	93
Specificity to SLE, %		81	91	94

*** Subject details:** 2 subjects had a stage III colorectal cancer: IIIA, T1, N1, M0; IIIC, T4b, N2, M0;

6 subjects had a malignant melanoma cancer, stages II (n= 2, stage IIA, T2b, N0, M0, and IIC, T4b, N0, M0), and III (n = 4; stage IIIA, T1a-4a, N1a, N2a, stage IIIb, T1b-4b, N1a, N2a, M0).

ANA status: ANA positivity was observed in 5 RA subjects (17%), 0 Crohn's disease, 0 APS, 0 cancer, 0 healthy controls. See Online methods for the details on ANA test.

Sensitivity to SLE was calculated as a ratio of positive samples for the corresponding assay to the total number of SLE diagnosed samples.[2] Specificity was calculated as a ratio of the positive signals across other diseases and heathy controls for the corresponding assay to the total number of control samples.

[2] Diagnoses and staging (for cancer) were given following recommended criteria, see Results in the main paper.

Table S8. ROC Table for ELISA and immunofluorescence assays (SSI non-matched cohort)*[3]

Signal cut-off	ELISA - Sensitivity, %	ELISA - Specificity, %	IFA - Sensitivity, %	IFA - Specificity, %
> 1,2	18	100	18	100
> 1	32	100	25	100
> 0,7	46	100	46	100
> 0.5	50	95	50	95

* HC was used for specificity calculation (n = 20, SSI)

[3] Caclulations were carried out as suggested in the literature: http://ebp.uga.edu/courses/Chapter%204%20-%20Diagnosis%20I/8%20-%20ROC%20curves.html

a)

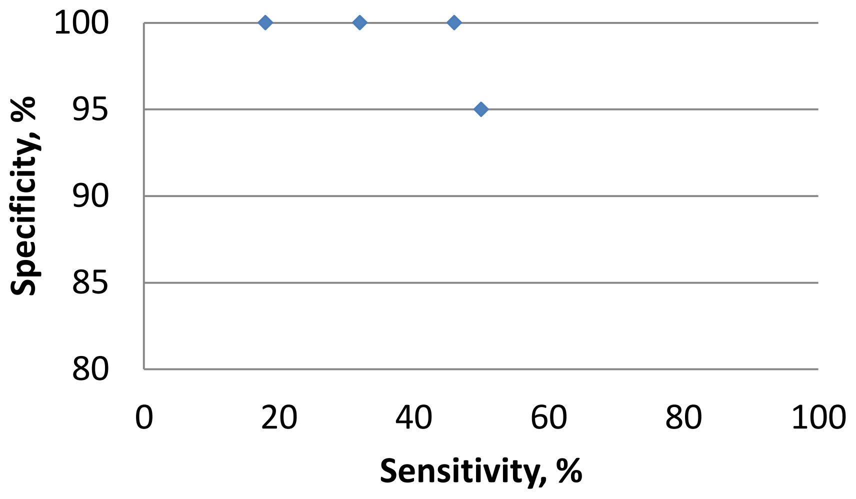

b)

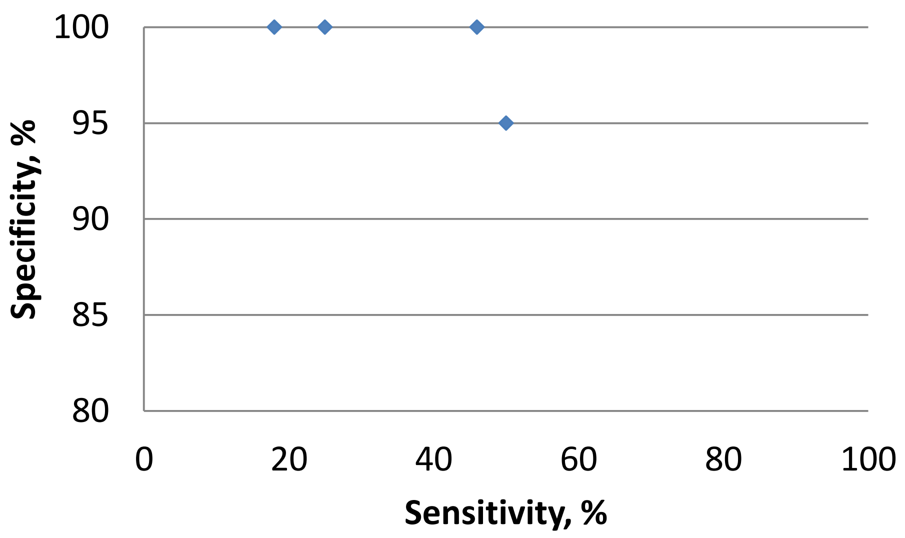

Supplemental Figure S6. ROC curves for non-matched SLE cohort (SSI) tested by SEQ1-ELISA (a) and TR-EG immunofluorescence assay (b). * Sensitivity and specificity are calculated based on the diagnosis SLE for 28 patients which has been given following SLEDAI calculation.
